# Supplementary material for: Association between oral microbiome and seven types of cancers in East Asian population: a two-sample Mendelian randomization analysis
Source: Front Mol Biosci. 2023 Nov 21;10:1327893. doi: 10.3389/fmolb.2023.1327893 (PMC10702768; doi:10.3389/fmolb.2023.1327893)
Supplement: Supplementary file 1 [file DataSheet1.ZIP › Supplementary Materials/MR plots for tongue/tongue═╝/Pancreatic cancer/╥╚╧┘░⌐_╔╕╤í╜ß╣√/pheno.150_to_pancreatic cancer_leave_one_out.pdf]

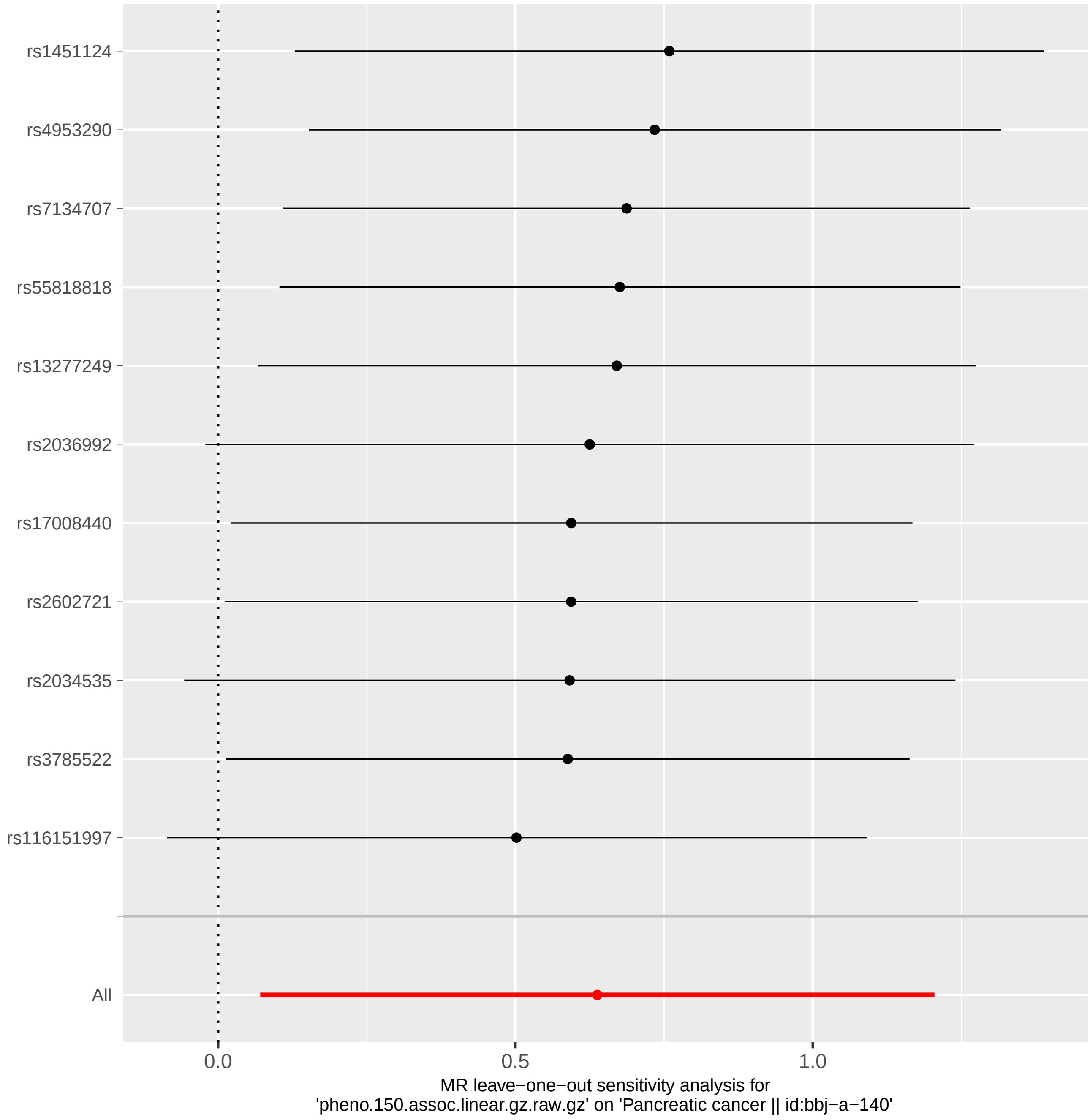
rs1451124
rs4953290
rs7134707
rs55818818
rs13277249
rs2036992
rs17008440
rs2602721
rs2034535
rs3785522
rs116151997
All
0.0
0.5
1.0
MR leave−one−out sensitivity analysis for
'pheno.150.assoc.linear.gz.raw.gz' on 'Pancreatic cancer || id:bbj−a−140'
